# Classification of Type 2 Diabetes Incidence Risk and the Health Behavior of the 30–50-Year-Old Korean Adults: Latent Class Analysis

**DOI:** 10.3390/ijerph192416600

**Published:** 2022-12-10

**Authors:** Roma Seol, Jin-Ho Chun

**Affiliations:** Department of Preventive Medicine, College of Medicine, Inje University, Busan 47392, Republic of Korea

**Keywords:** type 2 diabetes, latent class analysis, KNHANES, 30–59 years old, customized management

## Abstract

This study aimed to categorize the risk of type 2 diabetes mellitus development (T2DD) in the 30–50-year-old (3050) Korean adults and establish a baseline framework of customized management to prevent the progression to diabetes. A total of 9515 participants were enrolled in the Korea National Health and Nutrition Examination Survey (KNHANES) 2016–2019. Latent class analysis (LCA) was performed based on the health behaviors that were obtained from the secondary data source and were considered to affect T2DD. The major results were compared by latent class, multinomial regression analysis was performed, and the predicted risk of T2DD was evaluated using a self-assessment tool for Korean adults. Data analysis was performed using SPSS (ver. 25.0) and Mplus (ver. 8.6). The latent classes were divided into four categories: negative abdominal obesity and high-risk health behavior (Class A) (28.2%), negative abdominal obesity and low-risk health behavior (Class B) (37.1%), positive abdominal obesity and high-risk health behavior (Class C) (10.7%), and positive abdominal obesity and low-risk health behavior (Class D) (23.9%). The predicted risk scores for T2DD were 6.27 (Class C), 4.50 (Class D), 3.58 (Class A), and 2.16 (Class B), with a higher score indicating a worse state. Significant differences were observed in the predicted risk of T2DD between the latent classes, and abdominal obesity increased the risk. When managing the 30s–50s Korean generation physical activity and abdominal obesity control are strongly recommended.

## 1. Introduction

Diabetes is one of the most important health issues worldwide, and it is a chronic disease that requires continuous management efforts, such as the appropriate treatment and prevention of the complications. However, despite a lot of effort being made to curb it, the prevalence of diabetes has continued to increase [1]. According to a recent study conducted by the International Diabetes Federation, the number of patients with diabetes worldwide has increased significantly from 151 million in 2000 to 537 million in 2021 (International Diabetes Federation, 2021). This situation is no exception in South Korea. The prevalence of diabetes in adults aged 30 years or older in Korea more than doubled from 2.23 million in 2006 to 4.94 million in 2018; according to the Diabetes Fact sheet of the Korean Diabetes Association, one in seven patients aged 30 years or older in Korea is diabetic [2]. Hence, attention is being paid to the increasing prevalence of diabetes among young people from their 20s to their 30s based on the national disease statistics prepared by the Health Insurance Review and Assessment Service in 2021; it has been recognized as a primary public health issue. The number of diabetes patients increased by 51.4% from 19,781 people in their 20s in 2016 to 29,949 people in their 20s in 2020. This does not include the 27.7% increase in the diabetes prevalence among all of the age groups, including 30.8% of those in their 30s. According to the Diabetes Fact Sheet, 20% of all diabetic patients were less than 40 years of age; another study using data from the National Health and Nutrition Examination Survey also pointed out that the prevalence of pre-diabetes is rapidly increasing in the younger age group [3].

In the case of diabetes, blood sugar control is more difficult to achieve in the younger age group compared with that in the older adult group [4], and insulin resistance and pancreatic beta-cell dysfunction are severe and rapidly worsening [5]. Considering the severity and trend of diabetes, it is important to understand the potential disease risk and develop customized interventions for preventive management, especially for the younger age group.

The importance of the early detection and continuous management of diabetes has been emphasized for a long time; more recently, the necessity of implementing these measures has been highlighted. Active management should focus on improving health behavior and strengthening the efficiency of preventive management rather than that of treatment-oriented management. Moreover, the management of it at a young age is advantageous and should be emphasized. In the recent disability-adjusted life years (DALY) studies conducted in Korea, diabetes ranked first in terms of DALY in 2008 and 2018 [6], and diabetes at a younger age increased the risk of a longer disease exposure and complications; therefore, its early detection and management are of utmost importance [7]. According to the previous studies, the overall mortality rate has decreased by 4%, the incidence of macrovascular complications has decreased by 3%, and the incidence of microvascular complications has decreased by 5% for every 1-year increase in age at the time of the first diagnosis of diabetes mellitus [8]. In addition, a recent study indicated the need to extend the management guidelines for the old age group to the younger age group by including the 18-to-39-year-old age group in the high-risk group for diabetes [9].

Genetic and environmental factors act in a complex manner at the onset of diabetes, and the increase in the prevalence of diabetes in the younger generation is caused by environmental factors rather than genetic factors [10]. The main environmental factors that play a role in the development of diabetes such as occupation and individual behavioral characteristics are smoking, drinking, lack of physical activity, obesity and sleep disorders, and disordered eating behavior in individuals [11,12,13,14,15,16]. In addition, excessive energy intake and a lack of physical activity, which are typical lifestyle changes following economic development and westernization, are directly related to obesity, which is the most important underlying risk factor for diabetes [17]. In a previous study that investigated adolescents with type 1 diabetes for 8 years, the males and females were closely related to the overweight group, indicating that the risk of diabetes was seven times higher in both of the sex groups compared with that in the normal weight group with a body mass index (BMI) of 30 kg/m^2^ or higher [18].

To prevent the increase in the risk of diabetes, especially in the younger generation, active lifestyle improvements and blood sugar management are needed; however, the implementation of these measures remains a challenge due to the time constraints, overconfidence in health, and indifference related to economic activities. As the individual health behavior is often multidimensionally linked to the onset of the disease, it is important to manage it based on this multidimensional understanding, especially in the diabetes management of the younger generation.

A latent class analysis (LCA), applied as an important research technique in this study, is a method of subdividing and categorizing a class based on a combination of health behavior characteristics of an individual, rather than the existing variable-centered method. It is called a “person-centered approach,” which distinguishes different groups based on their experience with each health behavior. It is possible to stratify the individuals into groups with similar health behaviors; hence, this approach can be used in various groups such as children, adolescents, obese adults, and older adults. It has been widely used to explore multiple behavioral patterns [19,20,21,22].

Therefore, this analysis method can be used for establishing an individual risk diagnosis and developing customized intervention programs that are centered on relatively weak factors as it enables the comprehensive classification of the patients by type and characteristic [23]. However, the studies related to diabetes using this method are limited to the level of predictive factors for diabetes [24]. In addition, approximately 45% of the adults with diabetes worldwide remain undiagnosed [25]. Various attempts have been made for the early screening and appropriate management of diabetic patients, including the United States (US) model (ADA tool) [26], the US screening score [27], the European model (Rotterdam model) [28], and the Asian model (Tsingtao risk index) [29], which have been developed to predict the risk of developing diabetes in various countries and races.

Therefore, using the Korea National Health and Nutrition Survey (2016–2019), the latent class analysis was conducted based on the major health behaviors of the 30s–50s Korean population to categorize the diabetes risk factors, review the health behavior and disease management characteristics of the classified potential group and compare [30] and observe the results of this study using a standardized diabetes risk prediction tool for the 30s–50s Korean population.

## 2. Materials and Methods

### 2.1. Study Design

**Figure 1:**
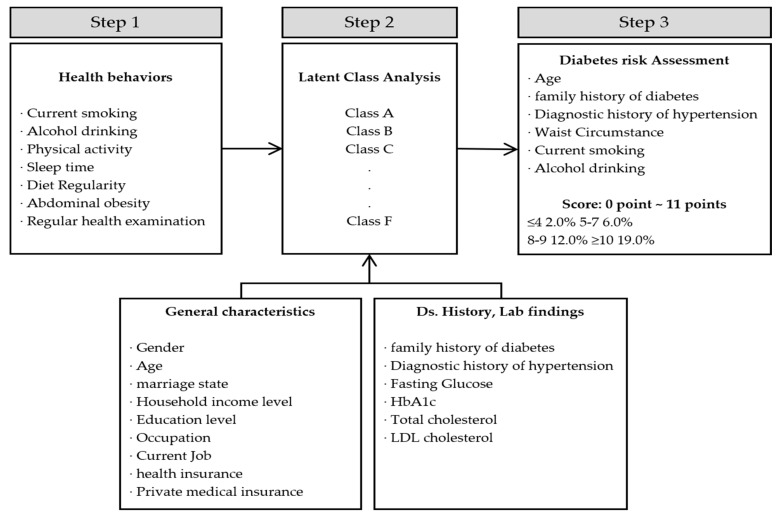
Flowchart of the study process.

To conduct a cross-sectional study using the data of 9515 people in their 30s –50s who participated in the Korea National Health and Nutrition Examination Survey (2016–2019), we developed a customized management framework by comparing and observing the management characteristics of the classified groups and the results of applying the diabetes risk prediction tool (Figure 1).

### 2.2. Study Participants

**Figure 2:**
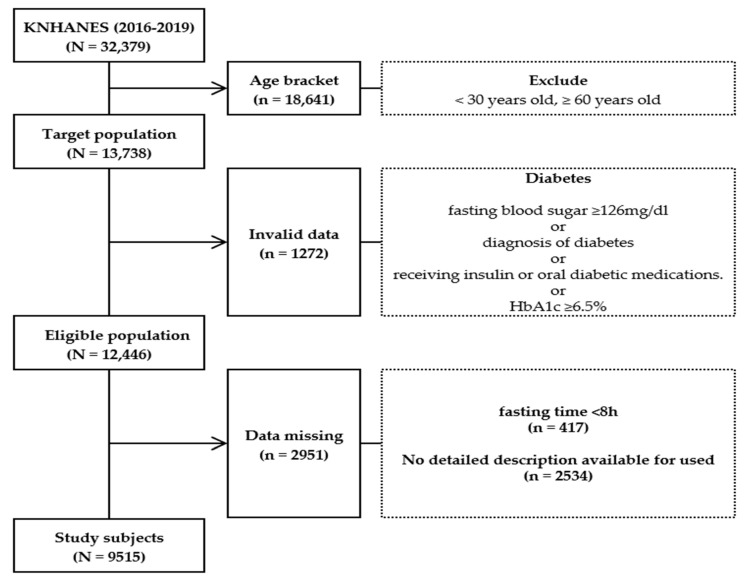
Flowchart showing the participant selection process.

Data of 32,379 people (8150 in 2016, 8127 in 2017, 7992 in 2018, and 8110 in 2019) were obtained from the Korea National Health and Nutrition Examination Survey (2016–2019) database, and this study included the participants in the study who were between the ages of 30–59 years. Among those who were aged between less than 30 years old and more than 60 years old or had a diagnosis history of diabetes or individuals who did not fast for 8 h prior to the blood sampling for the study were excluded. A total of 9515 subjects were selected (Figure 2).

The specific exclusion criteria applied when selecting the final study participants were as follows:

(1) Age: Individuals who were less than 30 years old and more than 60 years old;
(2) Nonconforming data: Individuals who did not fast for 8 h prior to blood sampling;
(3) Deficient data: Individuals whose fasting blood glucose and glycated hemoglobin test results were unavailable;
(4) Missing data.

### 2.3. Observational Variable

Gender, age, marital status, household income, education level, occupation, health insurance type, and private medical insurance were selected as the general characteristics, while current smoking status and drinking at least once a month were chosen as the health behavioral characteristics. The amount of aerobic physical activity was determined by substituting the MET-minute calculation formula of the International Physical Activity Questionnaire (IPAQ) [31] with 2 h and 30 min of moderate-intensity physical activity or more than 1 h and 15 min of high-intensity physical activity alone or moderate-intensity and high-intensity physical activities. Physical activity was defined as a combination of 1 min of high intensity and 2 min of moderate intensity activities. According to physical activity, the study participants were divided into a group that practiced the time equivalent to each activity and a group that did not. Sleep duration was defined as less than 7 h and more than 7 h, while the intake of regular meals was divided into irregular and regular meals according to their eating habits. With regard to the meal groups, the participants were classified according to the frequency of breakfast, lunch, and dinner within one week during the recent 1-year dietary survey; the meal frequency was rated as 5–7 times a week, 3–4 times a week, 1–2 times a week, and rarely (0 times a week). The regular meal group was defined as the group that skipped meals less than once a day. The participants whose frequency of eating breakfast, lunch, and dinner was 5–7 meals per week, and whose maximum frequency of missing meals per week was six times were classified as the regular meal group, while those who did not satisfy these criteria were classified as the irregular eating group [32].

Obesity and abdominal obesity were evaluated according to the guidelines of the Korean Society for Obesity [33]. The participants were classified as underweight, normal weight (<23.0 kg/m^2^), overweight (23.0–24.99 kg/m^2^), and obese (≥25.0 kg/m^2^) based on their BMI, while abdominal obesity was defined as a waist circumference of 90 cm or more for men and a waist circumference of 85 cm for women. The respondents were asked to respond with either “yes” or “no” to the question related to the health examination status based on whether or not they underwent a health examination in the last 2 years.

With regard to their medical history and clinical characteristics, diabetes management is currently applied to Koreans with an empty blood sugar level of 110 mg µg/kg dl and a 120-min postprandial blood sugar level of 200 mg µg/kg dl, which is based on a study that was presented by the Korean Diabetes Association [34]. In this study, diabetes was defined as a fasting blood sugar level of 126 mg/dL, a previous diagnosis of diabetes, the use of insulin or oral antidiabetes medications, or a glycated hemoglobin level of 6.5% or more, according to a recent study conducted by the Diabetes Fact Sheet in Korea 2020 and national health surveys [2]. A fasting blood sugar level of >100 mg/dL was classified as a fasting blood sugar disorder. Having a family history of diabetes was defined as a case in which either the father or both of the parents were diagnosed with diabetes. According to the standards of the Korean Society of Hypertension, hypertension was defined as a systolic blood pressure of 140 mmHg or higher, a diastolic blood pressure of 90 mmHg or higher, or the use of antihypertensive drugs [35]. Finally, the blood lipid level was classified as “appropriate” (total cholesterol: <200 mg/dl), “boundary” (total cholesterol: 200–239 mg/dl), or “risk” (total cholesterol: 240 mg/dl or more) according to the standards of the Korean Geological and Arteriochemical Association. High-density lipoprotein cholesterol (HDL) was classified as follows: >60 mg/dl is “appropriate”, 40–60 mg/dl is “alert”, and <40 mg/dl is “risk” for men, and >60 mg/dl is “appropriate,” 51–59 mg/dl is “alert,” and <50 mg/dl is “risk” for women. Neutral fat was defined as a triglyceride (TG) level of ≥150 [36].

The globally developed evaluation tool for predicting the risk of developing diabetes commonly includes the above indicators such as age, BMI, waist circumference, physical activity, daily vegetable, fruits intake, a diagnosis of hypertension, and having a history of drug use [27,37]. In particular, the history of hypertension diagnosis, hypertension, and diabetes are risk factors for cardiovascular disease, and they are important risk factors that cannot be excluded from the diabetes risk factors because of the high incidence of diabetes in hypertensive patients [38].

### 2.4. Statistics

The data analysis was performed using the SPSS ver. 25.0 (IBM, New York, NY, USA) and Mplus version 8.6 (Muthen & Muthen, Los Angeles, CA, USA), a specialized program for latent hierarchical analysis.

To identify the participants’ general characteristics, health behavior, disease history, and clinical examination or trial characteristics, the categorical data were analyzed by rate (%), while the continuous data were analyzed as mean and median, standard deviation, and range (from minimum to maximum). A latent class analysis was performed to classify the potential groups using the participants’ health behavior as the main factor variable. The “latent layers that were aggregated by similarity” were demarcated. The model fit was judged according to the Akaike information criterion (AIC) and Bayesian information criterion (BIC); these analytical methods emphasize the individuals included in the type [19]. Through the application of the Lo–Mendell–Rubin test, it was confirmed that the number of classified layers was the optimal value, and the accuracy of classification was determined by checking the entropy. Because the groups classified through the latent class analysis had different characteristics, a multinomial logistic regression analysis was performed to predict the group based on the general characteristics and compare the probability of belonging to the reference group. A multinomial logistic regression analysis allows for three or more categories in the modeled variable, and it is used to predict the probability of category membership on a dependent variable based on several independent variables [39].

## 3. Results

Of the total participants, 30.3% of them were in their 30s, 35.6% of them in their 40s, and 34.4% of them in their 50s, and 59.3% of them were women. Approximately 83.6% of the participants were married. In terms of income status, 6.8% of the participants belonged to the low income class, 22.4% of them belonged to the lower middle income class, and 29.2% of them belonged to the lowest income class. According to the level of education, 54.6% of the participants were college graduates, while 35.1% of them were high school graduates. The participants occupations were: managers (21.0%), office workers (16.6%), and service and sales workers (15.0%); meanwhile, 25.8% of them were unemployed, such as students and housewives. The types of health insurance were workplace insurance (71.6%) and community-based insurance (26.3%), while almost all (92.9%) of the participants had private health insurance. The main health behaviors of the participants were as follows: smoking (19.1%), drinking at least once a month (59.5%), not engaging in aerobic physical activity (54.9%), sleeping less than 7 h (48.1%), irregular eating (50.6%), a BMI of 25 kg/m^2^ or more (34.1%), abdominal obesity (waist circumference: ≥90 cm for male and ≥85 cm for female; 27.2%), and going without a medical examination (27.4%). The medical history and clinical examination characteristics included a family history of diabetes (26.4%), a history of hypertension (20.0%), fasting glucose disorder (31.8%), an uncontrolled blood sugar level (6.2%), a high cholesterol level (12.4%), an increased HDL level (29.3%), a high triglyceride level (29.6%), and metabolic syndrome (22.4%).

### 3.1. Latent Class Analysis

#### 3.1.1. Determining the Number of Latent Classes

In this study, a latent class analysis of the smoking, drinking, physical activity, regular meals, sleep time, BMI, waist circumference, and health examination data was conducted. The number of latent classes was finally determined by sequentially increasing the number of classes. AIC and BIC were used as fitness indices; the smaller the value was, then the more appropriate the model was judged to be. For each model, entropy, a value that is used to evaluate whether a target is accurately classified into each latent layer, was assessed; the closer it is to one, then the higher the classification accuracy is. Based on the results of the analysis, they were first classified into between two and five latent layers; the AIC and BIC values decreased as the number of latent layers increased. Therefore, the classification results of the final four potential layers were selected (Table 1).

#### 3.1.2. Differential Classification and Naming of Latent Classes

**Figure 3:**
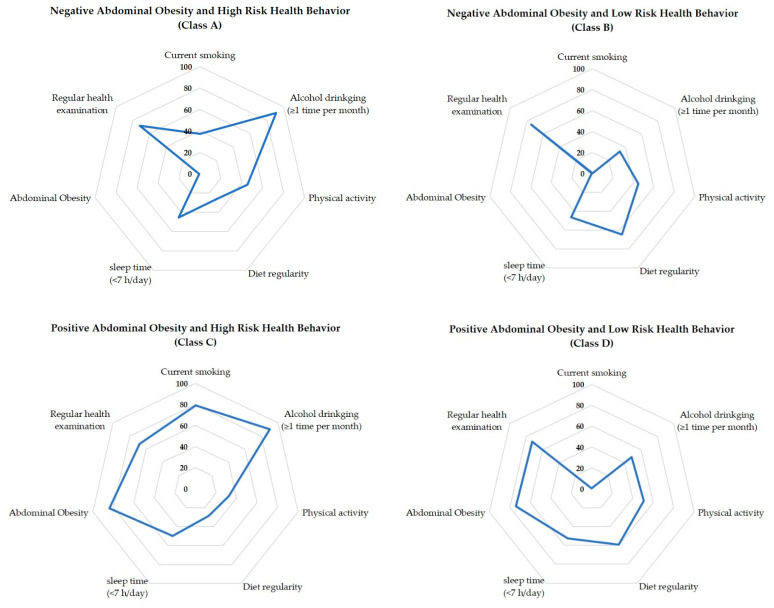
Item-response probabilities of health behaviors for the four-class model.

Based on the conditional probability values for major health behaviors, obesity, and health examination, the four potential classes selected as a result of the latent class analysis were no abdominal obesity/high-risk health behaviors (Class A), no abdominal obesity/low-risk health behaviors (Class B), high-risk health behaviors with abdominal obesity (Class C), and low-risk health behaviors with abdominal obesity (Class D) (Figure 3).

The Class A group had the lowest prevalence of abdominal obesity (0.5%), were current smokers (37.3%), consumed alcohol once a month (91.2%), performed a lack of physical activity (54.5%), and had irregular eating habits (71.9%), accounting for 28.2% of the total study population. The Class B group had almost non-existent abdominal obesity (0.7%), discontinued smoking (100%), consumed alcohol (66.4%), had regular eating habits (64.9%), and underwent health examinations (74.6%), accounting for 37.1% of the total study population. The Class C group was accompanied by abdominal obesity (83.6%), were current smokers (78.8%), consumed alcohol once a month (90.0%), performed a lack of physical activity (67.8%), and did not undergo health examinations (32.1%), accounting for 10.7% of the total study population. The Class D group was accompanied by abdominal obesity (74.4%), discontinued smoking (99.4%), consumed alcohol (51.3%), performed physical activities (50.9%), had regular eating habits (59.4%), and underwent medical examinations (72.6%), accounting for 23.9% of the total study population.

Among the four groups, the Class C group had the worst health behavior characteristics, which included smoking, drinking alcohol, a lack of physical activity, and irregular eating habits. In the Class B group, abdominal obesity was less prevalent, and the health behavior characteristics were generally healthy (Table 2).

#### 3.1.3. Comparison of Characteristics by Classified Latent Classes

The characteristics of the latent classes were compared according to the general characteristics of the participants (Table 3). In the Class A group, 54.2% of the participants were male, and the age range was 40–49 years (37.2%). Approximately 80.4% of the participants were married; 32.9% and 39.3% of them belonged to the middle income class and high income class, respectively, and 55.7% of them finished college or a had a higher degree. In terms of occupation, 21.3% of the participants were managers, 18.0% of them were office workers, and 15.5% of them were service workers. With regard to the types of health insurance, 70.5% of them had workplace insurance, while 93.2% of them had private health insurance. The Class B group had the highest proportion of female participants (81.8%). Approximately 37.4% of the patients in this group were aged 50–59 years, 85.7% of them were married, 30.7% them had a medium-to-high income, and 41.7% of them had a high income. Moreover, 58.6% of the participants finished college or had a higher degree. Their most common occupations were managers (22.3%), office workers (16.3%), and service workers (13.2%). The types of health insurance were workplace insurance (74.6%) and private health or medical insurance (93.3%). The Class C group had the highest proportion of male participants (82.6%). Approximately 36.1% of the patients belonged to the age groups of 30–39 years and 40–49 years, 78.7% of them were married, 35.7% of them had a medium-to-high income, and 52.5% of them finished college or had a higher degree. The most common occupations were functional workers (22.4%), managers (20.9%), and office workers (20.8%), and 87.8% of the patients comprised the occupational group. The types of health insurance were workplace insurance (66.2%) and private medical insurance (91.6%). In the Class D group, 59.2% of them were women, and 41.9% of them were aged 50–59 years. Moreover, 86.4% of the participants were married, 32.7% of them belonged in the lower-to-upper middle class, and 34.8% of them belonged in the upper middle class. Approximately 48.0% of them finished college or had a higher degree. The most common occupations were managers (18.7%), service workers (17.1%), and office workers (13.5%), and 73.1% of the patients comprised the occupational group. The types of health insurance were workplace insurance (70.6%) and private medical insurance (92.4%).

The characteristics of the classified latent strata were compared according to the disease, medical history, and clinical characteristics (Table 4). The Class A group had a family history of diabetes (25.5%) and were diagnosed with hypertension (14.8%), a high blood pressure (22.3%), an uncontrolled blood sugar level (3.7%), an impaired fasting glucose level (28.3%), a high total cholesterol level (10.5%), a low HDL level (21.3%), a low low-density lipoprotein (LDL) level (9.2%), a low TG level (27.6%), and metabolic syndrome (9.1%). The Class B group had a family history of diabetes (25.1%) and were diagnosed with hypertension (11.7%), high blood pressure (16.0%), an uncontrolled blood sugar level (3.3%), fasting blood sugar disorders (19.3%), a low total cholesterol level (10.4%), a low HDL level (24.8%), a low LDL level (9.1%), a low TG level (14.3%), and metabolic syndrome (5.8%). The Class C group had a family history of diabetes (30.7%) and were diagnosed with hypertension (34.9%), high blood pressure (41.5%), uncontrolled blood sugar (12.8%), fasting blood sugar disorders (54.5%), a low total cholesterol level (15.8%), a low HDL level (39.6%), a low LDL level (14.5%), a low TG level (60.9%), and metabolic syndrome (34.5%). The Class D had a family history of diabetes (27.4%) and were diagnosed with hypertension (32.5%), high blood pressure (62.1%), uncontrolled blood sugar (10.8%), fasting blood sugar disorders (45.2%), a low total cholesterol level (16.3%), a low HDL level (41.1%), a low LDL level (14.1%), a low TG level (41.6%), and metabolic syndrome (25.2%).

### 3.2. Multinomial Logistic Regression Analysis: Relationship between General Characteristics and Classified Latent Classes

The multinomial logistic regression analysis was performed on all of the four potential classes grouped by setting with Class B, the healthiest state, acting as the reference group and using the participants’ characteristics such as gender, age, income status, education level, economic activity, marital status, and family history as independent variables (Table 5). Based on the characteristics of Class B, the probability of belonging to Class A was higher for the men (OR = 5.69), the 30 year age group (OR = 2.63), the low education level group (OR = 3.11), and the current economically active group (OR = 1.16). The probability of belonging to Class C was higher among the male group (OR = 23.32), the 30 year age group (OR = 2.82), the low income group (OR = 1.91), the low education level group (OR = 3.29), and the current economic activity group (OR = 1.32). The probability of belonging to Class D was higher among the male group (OR = 3.48), the low income group (OR = 1.38), the low education level group (OR = 3.39), the married group (OR = 1.19), and the group with a family history of diabetes (OR = 1.35).

### 3.3. Application and Evaluation of Diabetes Risk Prediction Tools

The type 2 diabetes risk prediction tool developed for Koreans was applied to the classified potential classes, and the results were compared. The average risk score for all of the participants was 3.56 points, and this was followed by 6.27 (Class C), 4.50 (Class D), 3.58 (Class A), and 2.16 (Class B).

A total score of five or higher was used as the criteria for screening the diabetic patients; Class C had the highest proportion of participants who obtained the highest score (88.5%), which was followed by the Class D group (43.0%), the Class A group (29.1%), and the Class B group (6.9%) (Table 6).

## 4. Discussion

This study is the first one to categorize the risk factors for diabetes in Korean individuals in their 30s–50s using the secondary data source of the Korea National Health and Nutrition Examination Survey. For the optimal preventive management of diabetes, it is necessary to consider that each individual’s risk factors are different and that these risk factors can be combined in various ways to affect them. In this study, the latent class analysis method was applied to identify the link between the potential classes in a manner that comprehensively considers the similarity and differentiation of the risk factors.

The study reported that the potential classes related to the risk of type 2 diabetes among Korean individuals in their 30s–50s were no abdominal obesity/high-risk health behaviors (Class A), no abdominal obesity/low-risk health behaviors (Class B), high-risk health behaviors with abdominal obesity (Class C), and low-risk health behaviors with abdominal obesity (Class D).

Although the participants were Korean individuals in their 30s–50s, 10.7% of the total study population were categorized as Class C, which was marked by presence of abdominal obesity and high-risk health behaviors. Considering that the participants of this study were a general healthy group who had never been diagnosed with diabetes, this categorical level is relatively high. Therefore, appropriate management resources should be provided in the younger age group; based on the results of previous studies, the high-risk group should be identified and managed early by conducting screening tests among the younger age group [40]. Class B, with no abdominal obesity and low-risk health behaviors, accounted for 37.1% of the total study population.

The characteristics of the group classified by the latent hierarchical analysis were grouped by their health behaviors and waist circumference values. This finding suggests that the management of abdominal obesity is very important for the preventive management of the risk factors of diabetes. The result of this previous study confirms that weight loss is the most important factor in preventing diabetes; for every 1 kg of weight loss, the risk of diabetes is reduced by 16% [41]. In order to prevent morbidity, a previous study suggested that the strategy should depend on the presence or absence of obesity [42]. This finding is similar to that of previous studies, which showed that a lack of physical activity is highly correlated with the risk of developing diabetes, especially in middle-aged people [30,43]; this finding is also consistent with the report of another study, which showed that the dietary patterns in obese men are closely related to the onset of diabetes [44].

The results of the multinomial logistic regression analysis using Class B, which is the categorical potential class of Korean individuals in their 30s–50s with the lowest risk of diabetes and was assigned as the reference group, showed that the impact on the diabetes risk was different depending on the characteristics of the target group, such as gender, age, income, education, economic level, spouse status, and diabetes family history. This finding suggests that the effect of these factors on the diabetes risk differs according to the characteristics of the target group. However, most of the previous intervention studies have collectively applied the standard program without distinguishing the characteristics of these classes. The development and operation of a customized management program that fully considers the characteristics of the complex and diverse risk factors for each latent class should be emphasized. In the 30s–50s group, it is important to develop an approach that takes into account the basic characteristics of the target group such as the diabetes history of the immediate family.

An attempt was made to apply the diabetes risk prediction tool to the classified potential classes and compare the results by class. Initially, the tool developed for the Korean adults which was developed by the Finnish Diabetes Association were applied together to compare the results. However, in the Korea National Health and Nutrition Examination Survey, the source of the data for this study, among the eight factors required for evaluation using the Finnish tool, the intake of vegetables and fruits and whether or not they exercised for more than 30 min were not assessed, and in particular, the BMI and waist circumference standards were not suitable for the Koreans. Hence, the tool developed for the Korean adults was applied [30].

The results showed that Class C was the worst one among the latent classes identified by the latent class analysis in terms of the health behavior characteristics, which was followed by Class A, Class D, and Class B; however, Class D was the worst one among the latent classes in terms of the diabetes prediction risk evaluated using this tool. This result suggests that the effect of abdominal obesity on the risk of diabetes development is greater than that of the other high-risk health behaviors. During the evaluation using this tool, the probability of having diabetes was 6% or higher; the rate corresponding to a total score of five or higher, which is the criterion that warrants a diabetes screening test, was 88.5% for Class C, which was significantly higher than those of the other groups. This finding was similar to the results of a previous study on the factors related to pre-diabetes among Korean adults [45]. This result was in agreement with that of a previous study, which showed that the more severe the abdominal obesity was, then the higher the risk was of developing fasting blood sugar disorder and type 2 diabetes [46,47,48].

Considering Korea’s unprecedented aging population, it is unsuitable to expect health improvements and quality of life improvements for a community without the participation of the 30s–50s group. Efforts to promote interest and strengthen the management of the 30s–50s generation are urgently needed using different strategies such as the one in this study.

The limitations of this study include the cross-sectional nature of the study, the inability to acquire additional information that was not included, and also, nutritional information that was not disclosed at the time of the study in the Korea National Health and Nutrition Examination Survey, and the failure to accurately reflect the weight measurements that were needed when we were analyzing the secondary data. However, since most of the existing studies evaluate the factors affecting the risk of developing diabetes target patients with diabetes or those with other chronic diseases, the future studies should involve the customized management of a large general health group, especially young people.

This study has the following significance. Firstly, by applying a new approach using the health behavior factors in adults in their 30s–50s, the groups were categorized based on their risk factors. Secondly, the characteristics closely related to the risk of developing diabetes in the 30s–50s generation, which were considered as the general healthy group, were identified: the presence of abdominal obesity and their health status or health behaviors. Thirdly, the predictive risk of diabetes for each characteristic group was evaluated by applying a tool that was developed for Koreans to the classified latent group. Fourthly, when one is managing chronic diseases in terms of the quality of the population, such as the rapid aging of the population and the population cliff in Korea, the urgency of managing the chronic diseases in the younger age group should be emphasized, and one should include strategies that are tailored to their needs. A method for preparing a basic framework is proposed. The results of this study can be used as a reference for community-based intervention and cohort studies, and related follow-up studies are warranted.

## 5. Conclusions

Using data from the Korea National Health and Nutrition Examination Survey (2016–2019), the latent class analysis method was applied to the generation of Koreans in their 30s–50s to categorize the diabetes risk factors and to compare and observe the predictors of diabetes risk by the latent group for customized management.

In this study, the potential class associated with the risk of type 2 diabetes in Koreans in their 30s–50s was classified into four groups, and their health behaviors, such as drinking, physical activity, and regular eating, especially abdominal obesity, significantly increased the risk of type 2 diabetes. These findings suggest that action strategies for increasing participation in physical activity among young people and managing abdominal obesity can be effective in lowering their risk of developing diabetes.

## Figures and Tables

**Table 1:** Model fit statistics of the LCA models.

Classes	AIC	BIC	Adjusted BIC	LMR	Entropy	Latent Class Prevalence (%)				
						1	2	3	4	5
2	84,395.078	84,516.808	84,462.785	0.0000	0.847	36.98	63.02			
3	83,894.685	84,080.861	83,998.237	0.0000	0.689	26.76	31.57	41.67		
4	83,700.155	83,950.776	83,839.552	0.0498	0.606	28.20	37.14	10.73	23.93	
5	83,618.400	83,933.467	83,793.642	0.2306	0.675	17.10	24.91	11.53	10.03	36.44

AIC, Akaike information criterion; BIC, Bayesian information criterion; LMR, Lo–Mendell–Rubin test.

**Table 2:** Prevalence of health behaviors in all four class models.

Characteristics	Classification	Class A (*n* = 2683)	Class B (*n* = 3534)	Class C (*n* = 1021)	Class D (*n* = 2277)
Current smoking	Yes	37.3	0.0	78.8	0.6
	No	62.7	100	21.2	99.4
Alcohol consumption (≥1 time per month)	Yes	91.2	33.6	90.0	48.7
	No	8.8	66.4	10.0	51.3
Physical activity	Yes	45.5	44.8	32.2	50.9
	No	54.5	55.2	67.8	49.1
Diet regularity	Yes	28.1	64.9	28.9	59.4
	No	71.9	35.1	71.1	40.6
Sleep time (h/day)	<7	45.6	46.4	49.8	52.8
	≧7	54.4	53.6	50.2	47.2
Abdominal obesity ^†^	Yes	0.5	0.7	83.6	74.4
	No	99.5	99.3	16.4	25.6
Regular health examination	Yes	71.8	74.6	67.9	72.6
	No	28.2	25.4	32.1	27.4
Naming		Negative Abdominal Obesity and High-Risk Health Behavior	Negative Abdominal Obesity and Low-Risk Health Behavior	Positive Abdominal Obesity and High-Risk Health Behavior	Positive Abdominal Obesity and Low-Risk Health Behavior

^†^ Waist circumstance: values 90 cm for men and ≥85 cm for women.

**Table 3:** Differences in the general characteristics of the four latent classes.

Characteristics	Classification	Class A	Class B	Class C	Class D	x^2^	*p*
Age (year)	30s	972 (35.2)	983 (27.8)	369 (36.1)	534 (23.5)	196.477	0.000
	40s	998 (37.2)	1231 (34.8)	369 (36.1)	788 (34.6)		
	50s	713 (26.6)	1320 (37.4)	283 (27.7)	955 (41.9)		
Gender	Men	1454 (54.2)	643 (18.2)	843 (82.6)	928 (40.8)	1686.131	0.000
	Women	1229 (45.8)	2891 (81.8)	178 (17.4)	1349 (59.2)		
Current Spouse	Yes	2158 (80.4)	3029 (85.7)	804 (78.7)	1968 (86.4)	62.081	0.000
	No ^†^	525 (19.6)	505 (14.3)	217 (21.3)	309 (13.6)		
Household income (quartile)	Low	175 (6.5)	224 (6.3)	90 (8.8)	161 (7.1)		
	L-Medium	571 (21.3)	750 (21.2)	229 (22.4)	578 (25.4)		
	M-High	882 (32.9)	1086 (30.7)	364 (35.7)	745 (32.7)		
	High	1055 (39.3)	1474 (41.7)	338 (33.1)	793 (34.8)		
Education (school)	≤Elementary	98 (3.7)	103 (2.9)	32 (3.1)	148 (6.5)	102.358	0.000
	Middle	167 (6.2)	184 (5.2)	74 (7.2)	172 (7.6)		
	High	924 (34.4)	1177 (33.3)	379 (37.1)	864 (37.9)		
	≥College	1494 (55.7)	2070 (58.6)	536 (52.5)	1093 (48.0)		
Occupation ^‡^	Managers	571 (21.3)	787 (22.3)	213 (20.9)	426 (18.7)	449.567	0.000
	Office workers	484 (18.0)	577 (16.3)	212 (20.8)	308 (13.5)		
	Service and sales worker	417 (15.5)	468 (13.2)	157 (15.4)	390 (17.1)		
	Agriculture	43 (1.6)	55 (1.6)	28 (2.7)	74 (3.2)		
	Functional	452 (16.8)	256 (7.2)	229 (22.4)	292 (12.8)		
	Simple labor worker	168 (6.3)	222 (6.3)	57 (5.6)	175 (7.7)		
	Unemployed	548 (20.4)	1169 (33.1)	125 (12.2)	612 (26.9)		
Current job state	Yes	2135 (79.6)	2365 (66.9)	896 (87.8)	1665 (73.1)	237.754	0.000
	No	548 (20.4)	1169 (33.1)	125 (12.2)	612 (26.9)		
Type of health insurance	Community	730 (27.2)	383 (23.7)	312 (30.6)	626 (27.5)	38.540	0.000
	Workplace	1891 (70.5)	2638 (74.6)	676 (66.2)	1608 (70.6)		
	Medicare	62 (2.3)	58 (1.6)	33 (3.2)	43 (1.9)		
Private health state	Yes	2500 (93.2)	3297 (93.3)	935 (91.6)	2104 (92.4)	4.672	0.197
	No	183 (6.8)	237 (6.7)	86 (8.4)	173 (7.6)		

^†^ Single-divorce/widowed; ^‡^ Managers, professionals, and related workers/office workers/service and sales workers/agriculture, forestry, and fisheries workers/functional, device, and machine (assembly) workers/simple labor workers/unemployed (housewives, students, etc.).

**Table 4:** Differences in the disease history and laboratory characteristics of the four latent classes.

Characteristics	Classification	Class A	Class B	Class C	Class D	Statistics	*p*
Family history of diabetes	Yes	685 (25.5)	886 (25.1)	313 (30.7)	624 (27.4)	χ^2^ = 14.96	0.002
	No	1998 (74.5)	2648 (74.9)	708 (69.3)	1653 (72.6)		
History of being diagnosed with hypertension	Yes	397 (14.8)	415 (11.7)	356 (34.9)	739 (32.5)	χ^2^ = 556.92	<0.001
	No	2286 (85.2)	3119 (88.3)	665 (65.1)	1538 (67.5)		
Blood pressure (≥130/85 mmHg)	Yes	597 (22.3)	566 (16.0)	424 (41.5)	862 (37.9)	χ^2^ = 499.60	<0.001
	No	2085 (77.7)	2967 (84.0)	597 (58.5)	1415 (62.1)		
HbA1c (%)	Mean ± SD	113.54 ± 13.96	111.23 ± 14.17	120.99 ± 14.71	120.00 ± 14.7	F = 246.00	<0.001
		76.37 ± 9.57	73.94 ± 9.13	82.31 ± 10.05	6 80.31 ± 9.91	F = 321.02	<0.001
	<6.5	2583 (96.3)	3418 (96.7)	890 (87.2)	2030 (89.2)	χ^2^ = 304.36	<0.001
	≥6.5	100 (3.7)	116 (3.3)	131 (12.8)	247 (10.8)		
Fasting glucose (mg/dl)	Mean ± SD	5.51 ± 0.67	5.51 ± 0.57	5.84 ± 0.84	8.82 ± 0.89	F = 157.18	<0.001
	<100	1923 (71.7)	2851 (80.7)	465 (45.5)	1248 (54.8)	χ^2^ = 698.10	<0.001
	≥100	760 (28.3)	683 (19.3)	556 (54.5)	1029 (45.2)		
		97.15 ± 20.27	94.36 ± 16.76	107.14 ± 25.03	104.67 ± 28.34	F = 137.74	<0.001

**Table 5:** Multinomial logistic regression analysis of the latent classes based on the general characteristics.

Variables		Class B (Reference)		
		Class A	Class C	Class D
Gender	Men vs. women	5.69 (5.03–6.44)	23.32 (19.15–28.39)	3.48 (3.06–3.95)
Age	30s vs. 50s	2.63 (2.26–3.05)	2.82 (2.28–3.49)	1.04 (0.89–1.21)
	40s vs. 50s	1.97 (1.71–2.26)	1.92 (1.57–2.34)	1.11 (0.98–1.27)
Household income quartile (ref. high)	Low	1.02 (0.80–1.30)	1.91 (1.38–2.65)	1.19 (0.93–1.51)
	Low-medium	0.97 (0.84–1.13)	1.27 (1.03–1.57)	1.38 (1.19–1.59)
	Medium-high	1.06 (0.93–1.21)	1.41 (1.17–1.70)	1.26 (1.11–1.44)
Education level (ref. college)	≤Elementary school	3.11 (2.27–4.27)	3.29 (2.06–5.25)	3.39 (2.55–4.52)
	Middle school	2.36 (1.84–3.02)	3.14 (2.24–4.38)	2.00 (1.57–2.54)
	High school	1.61 (1.43–1.83)	2.03 (1.71–2.42)	1.56 (1.38–1.77)
Currently employed	Yes vs. no	1.16 (1.02–1.32)	1.32 (1.05–1.67)	0.98 (0.87–1.12)
Currently married	Yes vs. no	1.10 (0.91–1.32)	1.48 (1.23–1.75)	1.19 (1.05–1.35)
Family history	Yes vs. no	1.10 (0.97–1.24)	1.22 (0.95–1.55)	1.35 (1.09–1.66)

**Table 6:** Diabetes risk score according to the latent classes.

Variables	Classification	Total	Class A	Class B	Class C	Class D	Statistics	*p*
Score ^†^	≤4	6608 (69.4)	1903 (70.9)	3290 (93.1)	117 (11.5)	1298 (57.0)	χ^2^ = 3202.51	<0.001
	5–7	2474 (26.0)	730 (27.2)	241 (6.8)	644 (63.1)	859 (37.7)		
	8–9	418 (4.4)	50 (1.9)	3 (0.1)	245 (24.0)	120 (5.3)		
	≥10	15 (0.2)	0 (0.0)	0 (0.0)	15 (1.5)	0 (0.0)		
	Mean ± SD (min–max)	3.56 ± 2.09 (3.52–3.60)	3.58 ± 1.71	2.16 ± 1.55	6.27 ± 1.59	4.50 ± 1.63	F = 2097.58	<0.001
Probability of having diabetes	≥6%	2907 (30.6)	780 (29.1)	244 (6.9)	904 (88.5)	979 (43.0)	χ^2^ = 2718.48	<0.001

^†^ Diabetes risk score for Korean adults (probability of having diabetes): ≤4 points (2%), 5–7 points (6%), 8–9 points (12%), and ≥10 points (19%).

## Data Availability

The data for this study were downloaded from the National Health and Nutrition Survey website (http://knhanes.cdc.go.kr/, accessed on 27 April 2021) in compliance with the procedure for using data sources which removed the personal identification information such as names and resident registration numbers of individuals participating in the Korea National Health and Nutrition Survey (2016–2019). The reported data are available from the Korea Disease Control and Prevention Agency (KDCA). However, these data are available with the approval of KDCA.
